# A Multifaceted Presentation of the Retrocorneal Membrane Following Intraocular Surgery: A Case Series

**DOI:** 10.7759/cureus.81948

**Published:** 2025-04-09

**Authors:** Ching Yee Leong, Wan Haslina Wan Abdul Halim, Mazaya Mahmud, Ainal Adlin Naffi, Chenshen Lam, Meng Hsien Yong, Geok Chin Tan, Mae-Lynn Catherine Bastion

**Affiliations:** 1 Department of Ophthalmology, Faculty of Medicine, Universiti Kebangsaan Malaysia, Kuala Lumpur, MYS; 2 Department of Ophthalmology, Hospital Canselor Tuanku Muhriz, Kuala Lumpur, MYS; 3 School of Optometry, Faculty of Medicine and Health Sciences, UCSI University, Kuala Lumpur, MYS; 4 Department of Ophthalmology, Faculty of Medicine, University Putra Malaysia, Selangor, MYS; 5 Department of Pathology, Faculty of Medicine, Universiti Kebangsaan Malaysia, Kuala Lumpur, MYS; 6 Department of Pathology, Hospital Canselor Tuanku Muhriz, Kuala Lumpur, MYS

**Keywords:** corneal transplant, epithelial downgrowth, epithelial ingrowth, fibrous downgrowth, retrocorneal membrane

## Abstract

The retrocorneal membrane is a rare but serious complication of intraocular surgery, presenting in varied forms. This case series highlights three patients who developed retrocorneal membranes following different ocular procedures. The first case involves a 67-year-old man with a history of right open globe rupture. He underwent multiple surgeries and developed corneal decompensation, for which Descemet stripping automated endothelial keratoplasty (DSAEK) was performed. He later developed a recurrent epithelial downgrowth (EDG) membrane. The second case presents a 79-year-old man who presented with a worsening vision to 3/60, one year after a complicated cataract surgery, due to a temporally growing fibrous downgrowth. The third describes a 70-year-old man who experienced blurred vision with visual acuity (VA) of 6/60, caused by a nasally growing fibrous downgrowth 10 months post-endothelial keratoplasty. All patients underwent retrocorneal membrane peeling combined with intracameral 5-fluorouracil (5-FU) treatment. This report discusses their clinical presentations, ocular imaging, treatment approaches, and histopathological findings. Retrocorneal membranes, particularly EDG, are difficult to manage and often result in poor visual outcomes despite treatment. While histopathology (HPE) remains the gold standard for diagnosis, clinical evaluation, anterior segment optical coherence tomography (AS-OCT), and regular follow-up are essential in guiding management. Further research is needed to improve the understanding and treatment of this rare condition.

## Introduction

Retrocorneal membranes encompass all membranes located behind the cornea, with their etiology, manifestation, and management being of significant concern to ophthalmologists. They are classified into several types: epithelial downgrowth (EDG), fibrous downgrowth, inflammatory membrane, retained host Descemet membrane, and detachment of Descemet’s membrane from the graft [1]. EDG is a rare but serious complication of intraocular surgery and trauma. It is characterized by the invasion of translucent epithelial cell membranes into the anterior chamber (AC) of the eye, posing management challenges and often leading to poor outcomes [2]. EDG should be distinguished from fibrous downgrowth, despite similarities in etiology, risk factors, complications, and management strategies [2]. Fibrous downgrowth is typically less aggressive and involves fibrovascular tissue infiltrating the AC. Both EDG and fibrous downgrowth most commonly occur after intraocular surgery, such as cataract surgery, glaucoma surgery, penetrating keratoplasty (PK), Descemet stripping endothelial keratoplasty, or ocular trauma [3]. These conditions can lead to severe complications, including corneal endothelial failure, permanent vision loss, and refractory glaucoma [4,5].

This case series highlights key diagnostic challenges, treatment approaches, and long-term outcomes in patients who developed retrocorneal membranes following intraocular surgeries, specifically Descemet stripping automated endothelial keratoplasty (DSAEK) and cataract surgery. DSAEK is a partial-thickness corneal transplantation in which the diseased Descemet membrane and endothelium are selectively removed while preserving the anterior corneal layers. Donor tissue, consisting of a healthy endothelium and a thin layer of posterior stroma, is inserted into the AC through a surgical incision. An air bubble is introduced into the AC to facilitate graft adherence and remove interface fluid. Venting incisions, created with a 15-degree blade, are typically made as four evenly spaced cuts in the mid-peripheral cornea, directed toward the graft-host interface.

## Case presentation

First case

**Figure 1:**
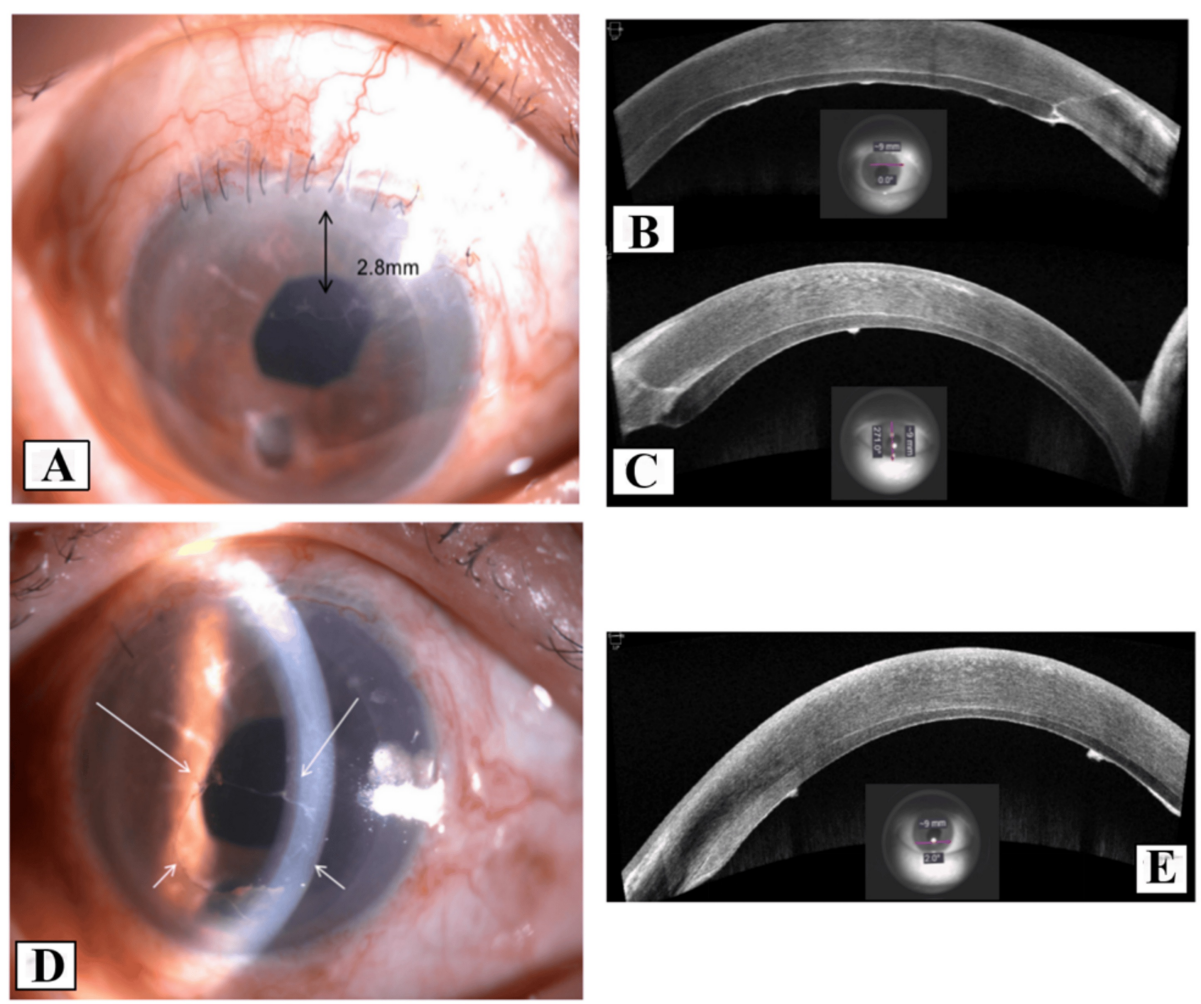
Ophthalmic examination of the first case (A) The 2.8-mm EDG involving the superior part of the posterior cornea (black arrow). (B) AS-OCT horizontal image of the superior cornea showing the hyperreflective EDG tissue. (C) Vertical image showing that the EDG grew toward the center of the cornea with smooth and rolled edges. (D) Anterior segment photo slit view showing EDG recurrence, which grew further toward the center of the cornea from 360 degrees (white arrow: margin of the membrane). (E) AS-OCT horizontal image at the inferior cornea showing the recurrence of hyperreflective EDG tissue. EDG: epithelial downgrowth, AS-OCT: anterior segment optical coherence tomography.

**Figure 2:**
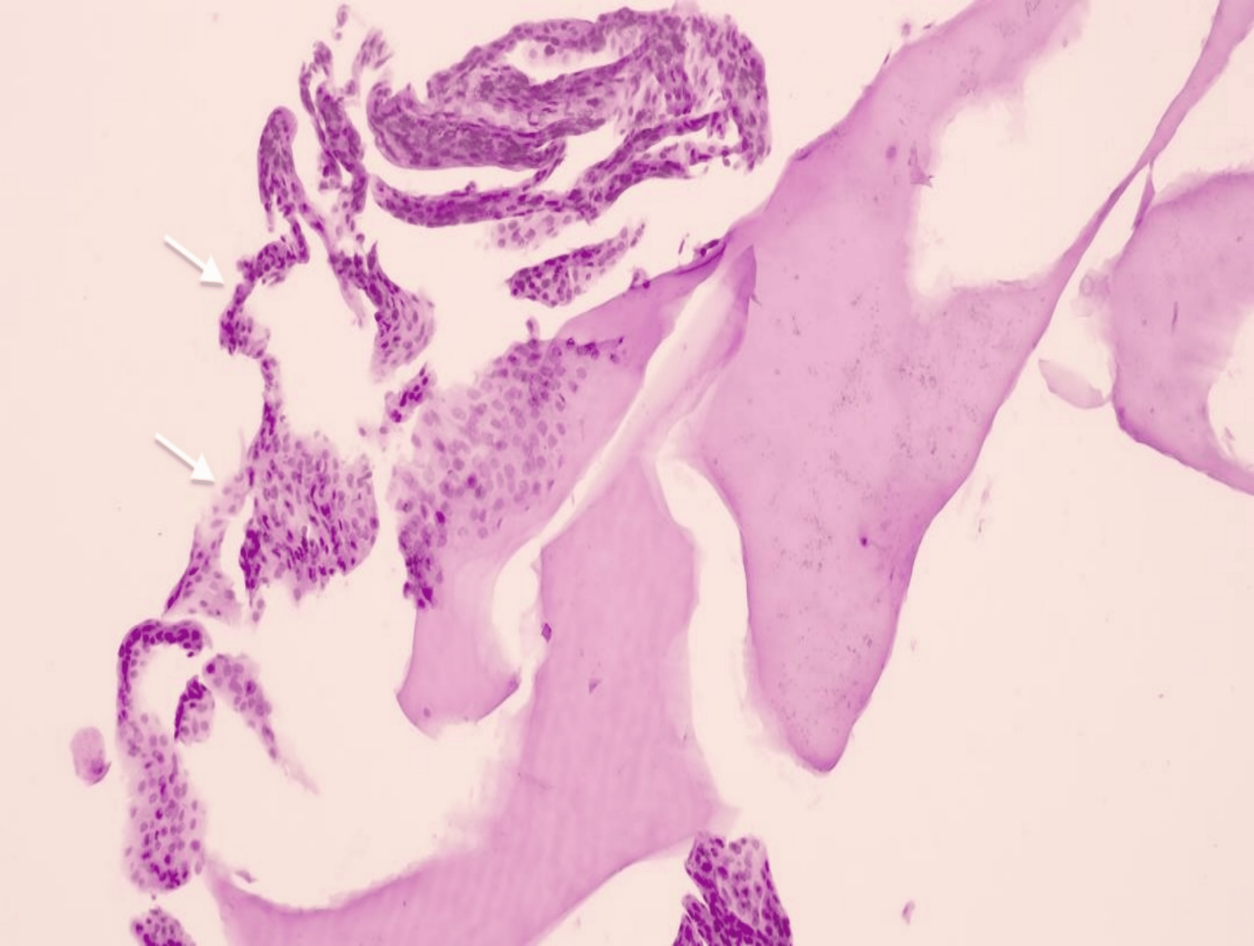
Epithelial downgrowth of the cornea White arrow showing nonkeratinized stratified squamous epithelial tissue (hematoxylin and eosin stain).

A 67-year-old Chinese man presented with a history of industrial trauma resulting in an open globe rupture and retinal detachment in the right eye (RE). He underwent multiple surgeries, including primary corneal and scleral toilet and suturing, followed by phacoemulsification with resultant aphakia for traumatic cataract, and pars plana vitrectomy with gas tamponade for retinal detachment. However, three months postoperatively, he developed corneal decompensation, with RE visual acuity (VA) declining from 6/60 to counting fingers (CF). One year later, he underwent RE DSAEK with scleral-fixated intraocular lens implantation. During the procedure, four venting incisions were made before Descemet stripping. The donor cornea was inserted using a glide, followed by intracameral air injection. The patient required two graft repositions and rebubbling after DSAEK. The surgery yielded a clear cornea with a VA of 6/24. Two months after DSAEK, a transparent retrocorneal membrane was observed superiorly, measuring 2.8 mm in diameter (Figure 1A), with VA stable at 6/24. A diagnosis of EDG was made based on clinical examination and AS-OCT (Figures 1B, 1C). Surgical peeling of the membrane was performed one month later, along with intracameral 5-FU (1 mg/0.1 mL) and 0.3 mL of Healon EndoCoat® Ophthalmic Viscoelastic (Johnson & Johnson, New Brunswick, USA). The membrane was excised as extensively as possible. However, 10 days postoperatively, a more aggressive EDG with pigment deposits was observed (Figures 1D, 1E), extending circumferentially toward the central cornea. A second surgical intervention was performed, including EDG peeling, endoscopic photocoagulation (ECP), and cryotherapy using a double freeze-thaw technique at -80°C, sparing the limbus. Intracameral 5-FU and Healon EndoCoat® were administered again. Histopathology (HPE) revealed multiple tiny fragments of corneal tissue composed of stratified squamous nonkeratinized epithelium, confirming the diagnosis of EDG (Figure 2). Unfortunately, a third recurrence was noted one month later. Due to financial constraints, the patient declined further intervention. The EDG progressively advanced, eventually covering the entire endothelium and leading to secondary glaucoma. The patient’s VA deteriorated to hand movements, accompanied by a positive relative afferent pupillary defect.

Second case

**Figure 3:**
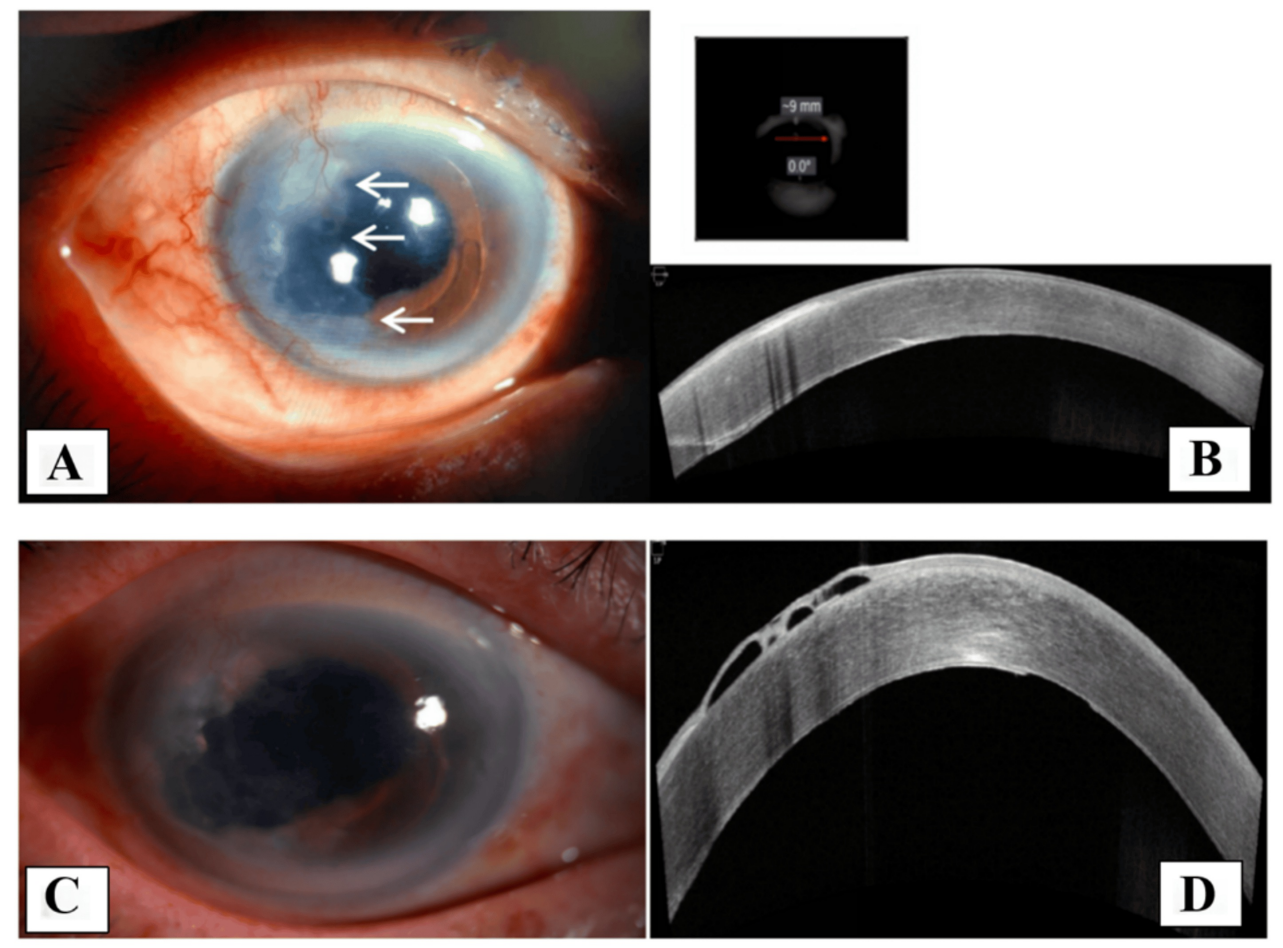
Ophthalmic examination of the second case (A) Anterior segment photo showing a thick retrocorneal membrane originating from the temporal side (white arrow: margin of the membrane). (B) AS-OCT image showing a membrane beneath the endothelium from the temporal aspect of the cornea. (C, D) Anterior segment photo and AS-OCT showing no regrowth of the membrane eight months postoperatively. AS-OCT: anterior segment optical coherence tomography.

**Figure 4:**
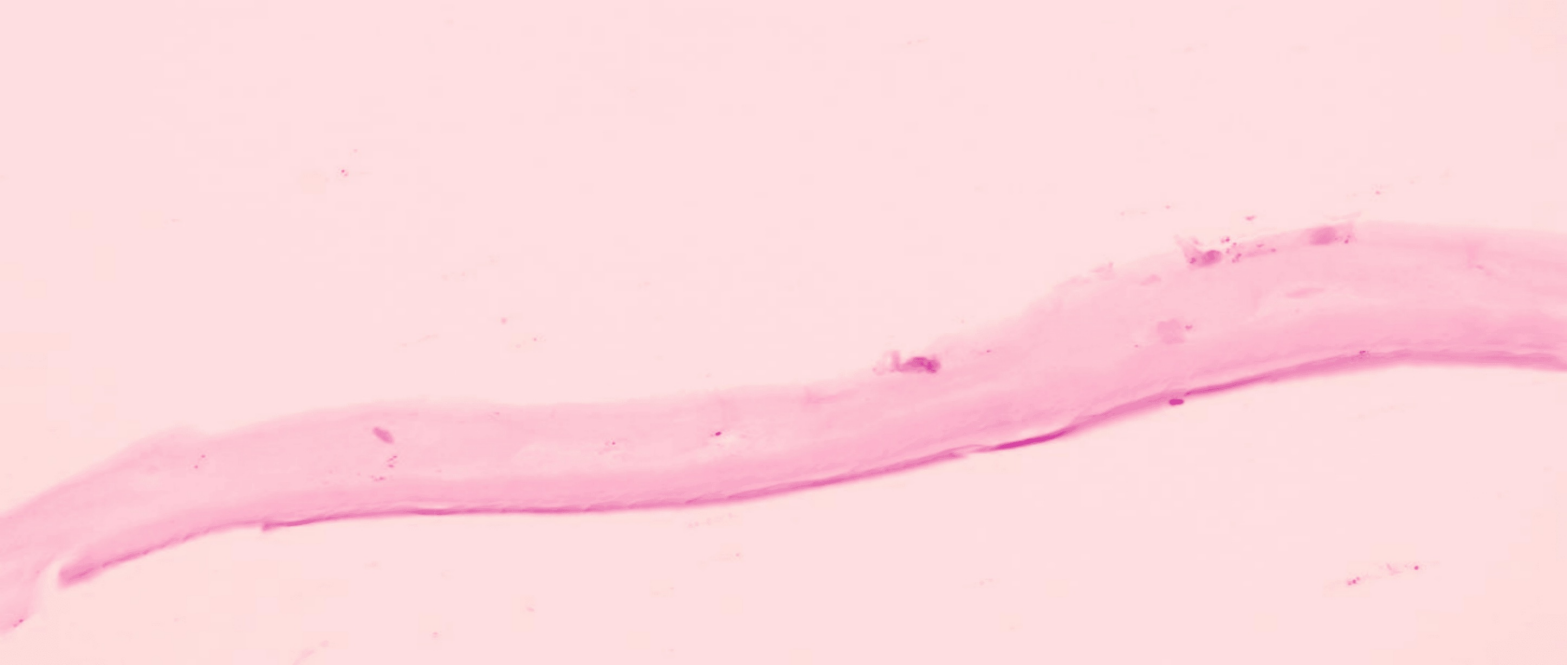
Fibrous downgrowth of the cornea Tissue composed mainly of fibroconnective tissue (hematoxylin and eosin stain).

A 79-year-old Chinese man with a history of RE phacoemulsification, which was converted to extracapsular cataract extraction (ECCE) via a temporal approach, presented with decreased vision after one year. Examination revealed a thick retrocorneal membrane originating from the temporal region with an irregular border (Figure 3A). VA was 3/60, and the intraocular pressure (IOP) was 16 mmHg. The clinical findings raised suspicion for either fibrous downgrowth or EDG. Anterior segment optical coherence tomography (AS-OCT) revealed a membrane located beneath the endothelium on the temporal side (Figure 3B). The patient underwent surgical peeling of the retrocorneal membrane, along with intracameral treatment with 5-FU (1 mg/0.1 mL) with 0.3 mL of Healon EndoCoat® Ophthalmic Viscoelastic. Postoperatively, no recurrence was observed after eight months (Figures 3C, 3D). VA improved to 6/24, and the patient remained stable thereafter. The HPE revealed benign fibroconnective tissue devoid of the epithelial lining, confirming the diagnosis of fibrous downgrowth (Figure 4).

Third case

**Figure 5:**
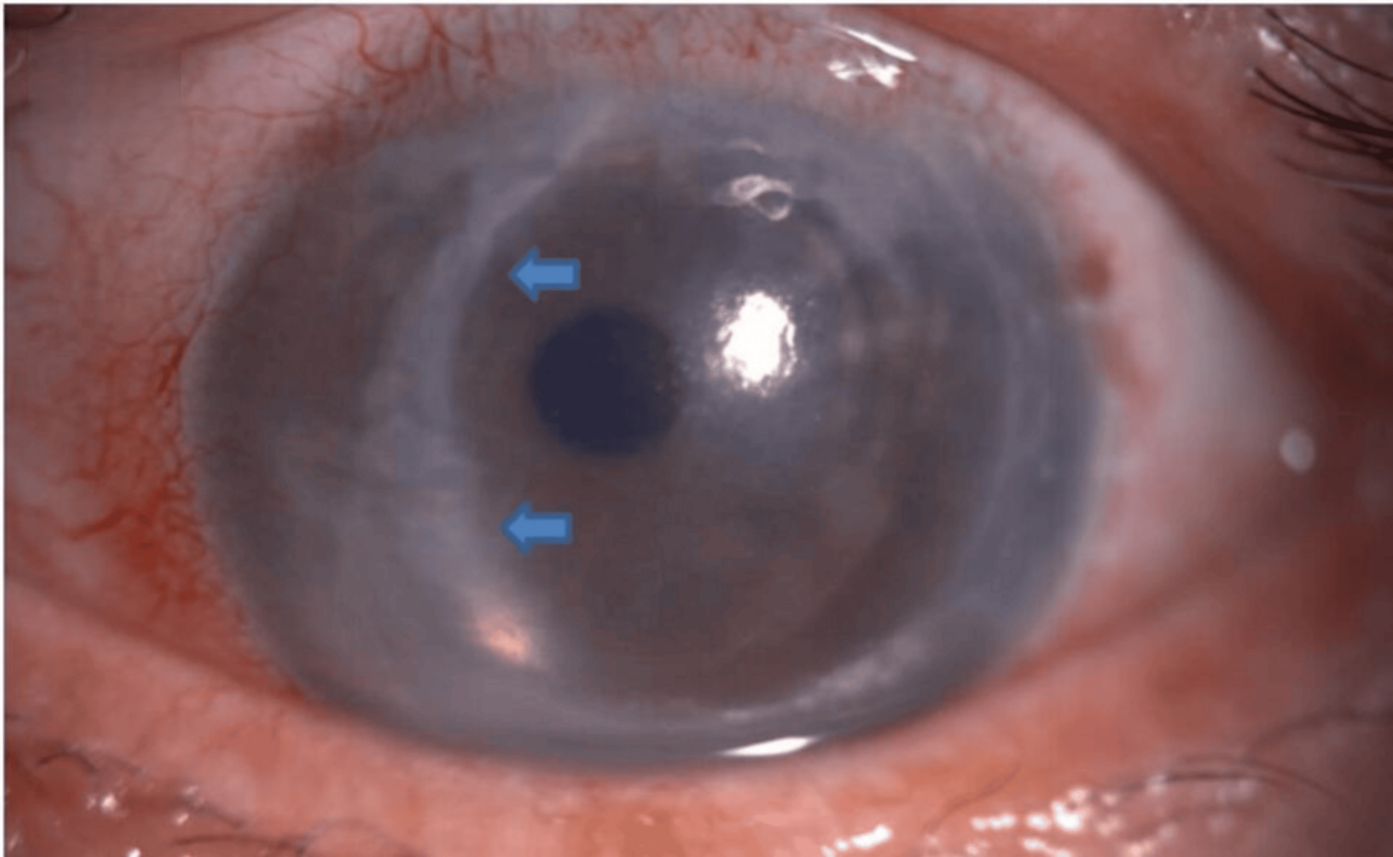
Ophthalmic examination of the third case Anterior segment photo showing the retrocorneal membrane 10 months after DSAEK (blue arrow: margin of the membrane).

**Figure 6:**
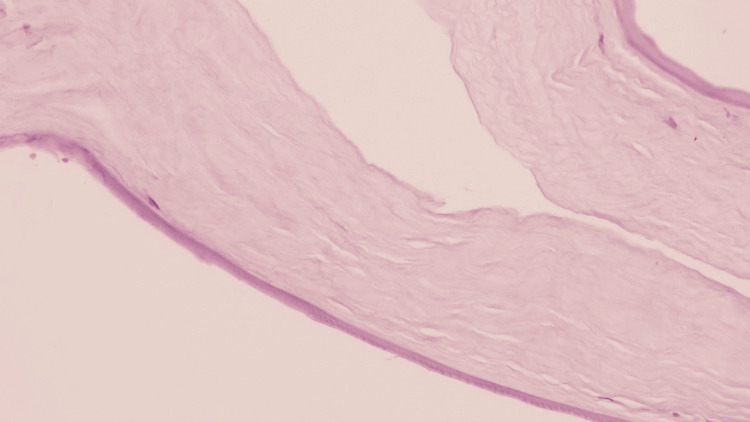
Fibrous downgrowth of the cornea Tissue composed mainly of loose fibroconnective tissue (hematoxylin and eosin stain).

A 70-year-old Malay man presented with pseudophakic bullous keratopathy after a complicated cataract surgery in which an anterior chamber intraocular lens (ACIOL) was implanted. He subsequently underwent DSAEK, which resulted in a clear cornea. However, 10 months after DSAEK, the patient reported reduced vision, with a VA of 6/60. Slit-lamp examination revealed a membrane on the nasal aspect of the posterior corneal surface (Figure 5). Based on clinical findings, a diagnosis of either EDG or fibrous downgrowth was considered. The patient was scheduled for downgrowth membrane peeling, DSAEK graft removal, ACIOL explantation, and intracameral administration of 5-FU (1 mg/0.1 mL) with 0.3 mL of Healon EndoCoat® Ophthalmic Viscoelastic. HPE analysis revealed benign fibroconnective tissue with no evidence of epithelial cells (Figure 6). Unfortunately, postoperative follow-up was not possible, as the patient passed away two weeks later due to a cardiac event.

Table 1 summarizes the patients’ surgical histories preceding the development of retrocorneal membranes, the interval between surgery and membrane onset, main features, diagnostic methods, treatment approaches, recurrence status, and visual outcomes.

**Table 1:** Case summary DSAEK: Descemet stripping automated endothelial keratoplasty, AS-OCT: anterior segment optical coherence tomography, EDG: epithelial downgrowth, ECP: endoscopic photocoagulation.

	First case	Second case	Third case
Age	67	79	70
Sex	Male	Male	Male
Surgical history	Primary cornea scleral toilet and suturing; phacoemulsificaltion + pars plana vitrectomy; DSAEK	Cataract extraction	Cataract extraction; DSAEK
Time from incident surgery	Two months after DSAEK	One year	Ten months after DSAEK
Main features	Retrocorneal membrane	Retrocorneal membrane	Retrocorneal membrane
Method of diagnosis	AS-OCT and HPE	AS-OCT and HPE	AS-OCT and HPE
Treatment	EDG peel with ECP, cryotherapy, and intracameral 5-FU (1 mg/0.1 mL) with 0.3 mL of Healon EndoCoat®	Membrane peeling and intracameral treatment with 5-FU (1 mg/0.1 mL) with 0.3 mL of Healon EndoCoat®	Membrane peeling, DSAEK graft removal, ACIOL explantation, and intracameral 5-FU (1 mg/0.1 mL) with 0.3 mL of Healon EndoCoat®
No. of downgrowth surgery	Two	One	One
Recurrence	Yes	No	Not assessed (the patient passed away)
Recurrence-free/follow-up period	-	Eight months	-
Visual outcome	Hand movement	6/24	Not assessed (the patient passed away)

## Discussion

EDG or fibrous downgrowth after intraocular surgery is rare. Common procedures associated with EDG include cataract extraction, penetrating keratoplasty (PK), DSAEK, Descemet membrane endothelial keratoplasty (DMEK), and glaucoma filtering surgery [3,6]. Several risk factors may predispose an eye to EDG, including multiple intraocular surgeries, inadequate wound closure, full-thickness suture tracts, chronic intraocular inflammation, tissue incarceration, stripping or damage to Descemet's membrane or corneal endothelium, and ocular hypotony [6]. In the first case of this series, multiple risk factors were present, including ocular trauma, multiple ocular surgeries, graft dislocations, and prolonged inflammation. It is possible that EDG developed prior to DSAEK, although this could not be definitively determined. Additionally, epithelial cell invasion may have occurred via the DSAEK venting incisions or during graft repositioning and rebubbling. Semeraro et al. identified three potential mechanisms for EDG following DSAEK: epithelial cell entry through full-thickness corneal incisions, the use of trephined grafts containing full-thickness tissue, and the inadvertent intracameral introduction of epithelial cells during surgery [7].

Most patients with EDG or fibrous downgrowth present within 6-12 months post-surgery or trauma, although reported cases have ranged from as early as 4 days to as late as 38 years postoperatively [3]. The common presenting symptoms include decreased vision, ocular redness, pain, and photophobia. Clinical signs such as corneal haze, intraocular inflammation, and elevated intraocular pressure can complicate the diagnostic process [5]. In the first case, post-traumatic corneal decompensation may have obscured the initial diagnosis of EDG. Fibrous downgrowth shares similar risk factors, symptoms, and clinical signs with EDG, but differs in behavior and appearance. Clinically, fibrous downgrowth is recognized as a retrocorneal membrane that may be vascularized and is typically self-limiting or slowly progressive, in contrast to the more aggressive nature of EDG [3]. EDG typically appears as a translucent sheet with a smooth, well-defined border and rolled edges, whereas fibrous downgrowth tends to be thicker with irregular borders [4], as observed in the second and third cases in this series.

The gold standard for diagnosis is histological assessment, which distinguishes between EDG and fibrous downgrowth, as well as the corneal or conjunctival origin of EDG. EDG usually manifests as multilayered, nonkeratinized, stratified squamous epithelial sheets, whereas fibrous downgrowth appears thicker due to the proliferation of fibrocytes [4]. The presence of goblet cells indicates that the invading epithelium is of conjunctival origin [6]. Prognostically, fibrous downgrowth has a more favorable outcome than EDG [4].

Argon laser photocoagulation also aids in diagnosing EDG. When applied to the overgrown epithelium on the iris, it produces a pathognomonic fluffy white reaction. In contrast, photocoagulation of the iris mesoderm without EDG does not elicit any observable change [2,8]. Noninvasive imaging modalities such as AS-OCT, specular microscopy, and confocal scanning are also useful in diagnosing EDG [9]. AS-OCT can detect hyperreflective retrocorneal tissue and plays an important role in identifying EDG. This noninvasive and noncontact imaging technique captures images at a speed of 40,000 A-scans per second, with an axial resolution between 3.9 and 7 µm. In some cases, EDG appears as a thin, hyperreflective layer along the posterior cornea [10], whereas fibrous downgrowth typically presents as a thicker, more irregular, and heterogeneous membrane. Confocal microscopy may provide additional cellular-level details. EDG is characterized by hyperreflective nuclei and multilayered epithelial cells at the endothelium [10], while fibrous downgrowth shows a hyperreflective, fibrous-appearing layer with activated keratocytes [11].

Treatment options for EDG and fibrous downgrowth vary based on disease progression and include surgical excision, cryotherapy, irradiation, laser photocoagulation, regrafting, and less invasive approaches such as intracameral injection of antimetabolites like 5-FU [5,8,12]. While some studies have reported successful arrest of EDG progression after 5-FU injections [4,13,14], others have shown limited or no effect [9,15]. There is no single definitive strategy for managing EDG, and treatment often involves complex surgical interventions. The first case was treated with surgical debridement, intracameral injection of 5-FU and viscoelastic, ECP, and cryotherapy. ECP enables direct visualization during membrane ablation, thereby minimizing collateral damage [16]. Intracameral 5-FU was used to eliminate residual epithelial cells; however, the EDG eventually recurred. Complete removal of epithelial sheets remains unachievable in many cases.

Intracameral 5-FU has shown efficacy in treating EDG by selectively targeting proliferating epithelial cells, with reported doses ranging from 0.04 to 1 mg per injection. Single injections may be inadequate due to the presence of quiescent cells, which can later become active and lead to recurrence. Sequential treatments at 2-3-week intervals have demonstrated improved clearance rates [17]. Kam et al. reported a 58% success rate with 5-FU monotherapy, while combination therapy with membranectomy achieved complete disease control [17]. However, concerns persist regarding potential corneal endothelial toxicity. Animal studies suggest a toxicity threshold between 1 and 10 mg/mL with a four-hour exposure, though clinical data remain inconclusive [16]. Notably, Wong et al. observed no significant endothelial cell loss one year after intracameral 5-FU (0.04 mg/0.1 mL) in a case of EDG following DSAEK, suggesting a dose-dependent safety margin [13]. Further research is needed to establish the optimal balance between therapeutic efficacy and endothelial safety.

Transcorneal cryotherapy is another treatment option for EDG. In the first case, a double freeze-thaw technique at -80°C was applied to the cornea, targeting the suspected site of invasion while sparing the limbus. This approach aims to maximize the destruction of the EDG while preserving limbal stem cells [18]. The second and third cases were managed under the presumption of EDG, though fibrous downgrowth remained a differential diagnosis. HPE ultimately provided the definitive diagnosis. In contrast to EDG, fibrous downgrowth is often managed medically, with treatment directed toward associated complications such as glaucoma, inflammation, and corneal edema. Surgical intervention may be required, though complete removal of fibrous tissue is generally not necessary [3]. 

However, our case series has several limitations. Follow-up was inconsistent across cases: the third case died before the first postoperative follow-up, and financial constraints in the first case prevented further treatment and long-term monitoring. Additionally, the small sample size limits the generalizability of our findings. Despite these limitations, this case series provides clinical insights into the diagnosis and management of retrocorneal membranes and highlights the utility of AS-OCT in characterizing these rare conditions.

## Conclusions

The evaluation and management of retrocorneal membranes remain challenging, particularly in cases of EDG, the most aggressive form, which is prone to recurrence and often results in poor visual outcomes despite aggressive treatment. While HPE remains the diagnostic gold standard, a comprehensive approach, including thorough clinical history, slit-lamp examination, AS-OCT imaging, and consistent follow-up, can aid in identifying the membrane type and guiding appropriate management. To improve long-term outcomes, further research is essential to better understand the disease process and establish more effective treatment strategies. Limitations of this case series include the small sample size and inconsistent follow-up, which may affect the generalizability of the findings.
